# A TGF-β type II receptor that associates with developmental transition in *Haemonchus contortus* in vitro

**DOI:** 10.1371/journal.pntd.0007913

**Published:** 2019-12-02

**Authors:** Li He, Robin B. Gasser, Tingting Li, Wenda Di, Fangfang Li, Hongrun Zhang, Caixian Zhou, Rui Fang, Min Hu

**Affiliations:** 1 State Key Laboratory of Agricultural Microbiology, Key Laboratory for the Development of Veterinary Products, Ministry of Agriculture, College of Veterinary Medicine, Huazhong Agricultural University, Wuhan, Hubei, China; 2 Melbourne Veterinary School, Department of Veterinary Biosciences, Faculty of Veterinary and Agricultural Sciences, The University of Melbourne, Parkville, Victoria, Australia; University of Calgary, CANADA

## Abstract

**Background:**

The TGF-β signalling pathway plays a key role in regulating dauer formation in the free-living nematode *Caenorhabditis elegans*, and previous work has shown that TGF-β receptors are involved in parasitic nematodes. Here, we explored the structure and function of a TGF-β type II receptor homologue in the TGF-β signalling pathway in *Haemonchus contortus*, a highly pathogenic, haematophagous parasitic nematode.

**Methodology/Principal findings:**

Amino acid sequence and phylogenetic analyses revealed that the protein, called *Hc-*TGFBR2 (encoded by the gene *Hc-tgfbr2*), is a member of TGF-β type II receptor family and contains conserved functional domains, both in the extracellular region containing cysteine residues that form a characteristic feature (CXCX_4_C) of TGF-β type II receptor and in the intracellular regions containing a serine/threonine kinase domain. The *Hc-tgfbr2* gene was transcribed in all key developmental stages of *H*. *contortus*, with particularly high levels in the infective third-stage larvae (L3s) and male adults. Immunohistochemical results revealed that *Hc-*TGFBR2 was expressed in the intestine, ovary and eggs within the uterus of female adults, and also in the testes of male adults of *H*. *contortus*. Double-stranded RNA interference (RNAi) in this nematode by soaking induced a marked decrease in transcription of *Hc-tgfbr2* and in development from the exsheathed L3 to the fourth-stage larva (L4) in vitro.

**Conclusions/Significance:**

These results indicate that *Hc-*TGFBR2 plays an important role in governing developmental processes in *H*. *contortus* via the TGF-β signalling pathway, particularly in the transition from the free-living to the parasitic stages.

## Author summary

*Haemonchus contortus* is a gastrointestinal parasitic nematode that causes major economic losses in small ruminants. Here, we investigated the structure and function of a TGF-β type II receptor homologue (*Hc*-TGFBR2) and its role in regulating *H*. *contortus* development. The results showed that the *Hc-tgfbr2* gene was transcribed in all developmental stages of *H*. *contortus*, with the highest level in L3s and male adults; the encoded protein *Hc*-TGFBR2 was expressed in the intestine and gonads of adult stages of this nematode. The transcriptional abundance of *Hc-tgfbr2* decreased significantly following knockdown by RNA interference in xL3s of *H*. *contortus*, which also caused a marked reduction in the number of xL3s developing to L4s in vitro. These findings reveal that the TGF-β type II receptor (*Hc-*TGFBR2) associates with development of *H*. *contortus*, particularly in its transition from the free-living to the parasitic stage.

## Introduction

In the free-living nematode *Caenorhabditis elegans*, the transforming growth factor β (TGF-β or DAF-7) signalling pathway is known to be centrally involved in regulating arrested development (dauer formation) [1, 2]. In this pathway, signals from the environment are first sensed by the TGF-β ligand, DAF-7 [3], which then binds to the TGF-β type II receptor DAF-4 [4, 5] in the cell membrane; then, the TGF-β type I receptor, DAF-1, is recruited and forms a ligand-receptor complex with the TGF-β ligand and TGF-β type II receptor [5, 6]. This compound transmits the signals by phosphorylating downstream R-Smad components, including DAF-8 and DAF-14, in the cytoplasm [7, 8]; subsequently, the activated R-Smad components enter the cell nuclei and inhibit the functions of Co-Smad (DAF-3) [9] and Sno/Ski (DAF-5) [10, 11], which promotes dauer formation. Although it has been proposed that arrested development (hypobiosis or diapause) in parasitic nematodes might be regulated in a similar way to dauer in *C*. *elegans*, there are significant knowledge gaps in relation to the former nematodes [12].

Nonetheless, some components of the TGF-β (DAF-7) signalling pathway have been studied in selected parasitic nematodes. For instance, the TGF-β ligand has been identified in *Ancylostoma caninum* [13, 14], *Haemonchus contortus* [15], *Teladorsagia circumcincta* [15], *Heligomosomoides polygyrus* [15], *Nippostrongylus brasiliensis* [15], *Parastrongyloides trichosuri* [16, 17], *Strongyloides ratti* [16], *Strongyloides stercoralis* [18] and *Brugia malayi* [19], suggesting that a TGF-β signalling pathway is present and/or active in these worms. In addition, a TGF-β receptor, *Bp-trk-1*, was reported for *Brugia pahangi* and contains the glycine-serine rich sequence (GS domain) of TGF-β type I receptors [20]. Recently, a TGF-β type I receptor-like molecule (*Hc-tgfbr1*) was characterised for *H*. *contortus* and inferred to be involved in the transition from the xL3 to the L4 stage [21]. In the present study, we explore the *C*. *elegans* homologue of a TGF-β type II receptor-like molecule encoded in *H*. *contortus* by a gene designated *Hc-tgfbr2*, and investigate its relevance and involvement in *H*. *contortus* development.

## Methods

### Ethics statement

Experimental goats used in this project were maintained in strict accordance with the Rules for Animal Ethics and Experimentation in the People’s Republic of China. The care and maintenance of goats were in accordance with protocols approved by The Scientific Ethics Committee of Huazhong Agricultural University (permit HZAUGO-2015-006).

### Maintenance of *H*. *contortus*

The Haecon-5 strain of *H*. *contortus* was maintained in experimental goats (3–6 months of age; raised helminth-free), which were infected orally with 8,000 third-stage larvae (L3s). Eggs were isolated from the faeces from infected goats as described previously [22]. First-stage (L1s), second-stage (L2s) and L3s of *H*. *contortus* were collected following 1, 4 and 7 days of copro-culture (28°C), respectively, washed several times in physiological saline and purified by migration through a nylon filter (mesh-size: 20 μm). The fourth-stage larvae (L4s) and adults of *H*. *contortus* were harvested from the abomasa of infected goats that were euthanised with an overdose of pentobarbitone sodium (Lethobarb, Virbac Pty. Ltd, Australia) at 8 and 30 days of infection, respectively. Female and male worms were separated and washed extensively in physiological saline, and then snap-frozen in liquid nitrogen and stored at -80°C.

### Preparation of nucleic acids

Using a Wizard DNA Clean-Up System (Promega Corporation, USA), genomic DNA was isolated from single female or male adults of *H*. *contortus*. Using the TRIzol reagent extraction method (Life Technologies, USA), total RNA was isolated from pools of 100–500 individuals of each developmental stage (i.e. egg, L1, L2, L3, female L4, male L4, female adult and male adult stages) of *H*. *contortus*. RNA yields were verified by spectrophotometric analysis (NanoDrop Technologies), cDNA was reverse transcribed from total RNA (1 μg) using the RevertAid First-Strand cDNA Synthesis Kit (Thermo Scientific, USA) to amplify coding sequence, or employing the PrimeScript RT reagent Kit with gDNA Eraser (Perfect Real Time) (Takara, Japan) for real-time PCR. RNA and DNA were stored at -80°C and -20°C, respectively.

### Genomic DNA and cDNA sequences of *Hc-tgfbr2*

From available transcriptomic datasets for *H*. *contortus* [23, 24], the coding sequence of *Hc-tgfbr2* (GenBank accession no. HF967728.1) was retrieved and used for the design of the primer pair Hc-tgfbr2-cF/cR (S1 Table), with which the inferred coding sequence of this gene was PCR-amplified from *H*. *contortus* cDNA using the following cycling conditions: 98°C/10 min, followed by 98°C/10 s, 55°C/15 s; 72°C/2 min for 35 cycles; and then 72°C/10 min. PCR was performed in a volume of 50 μL using 750 ng of cDNA, 0.2 μM of each forward primer and reverse primers (Hc-tgfbr2-cF/cR, S1 Table) and PrimeSTAR Max Premix (Takara, Japan), as recommended by the manufacturer (Takara). A no-cDNA control was included. Subsequently, the PCR product was cloned into the pTOPO-Blunt Simple vector (Aidlab Biotechnologies Co., Ltd). Two pairs of primers, Hc-tgfbr2-gF1/gR1 and Hc-tgfbr2-gF2/gR2 (S1 Table), were designed to amplify the two gap sequences from the *Hc-tgfbr2* genomic DNA region [23, 24]. Two “gap sequences” were partial regions of the *Hc-tgfbr2* genomic sequence that contained “Ns” in published genomic data for *H*. *contortus* [23, 24]. The two gap sequences were PCR-amplified from 300 ng *H*. *contortus* genomic DNA in 50 μL using 0.4 μM of each forward primer and reverse primers (S1 Table), 0.2 mM each of dNTP and 1 U Phanta Super-Fidelity DNA Polymerase (Vazyme Biotech Co Ltd, China), as recommended by the manufacturer (Vazyme) under the following cycling conditions: 95°C/5 min, followed by 95°C/30 s, 55.4°C/30 s; 72°C/8 min for 35 cycles; and 72°C/5 min, and then cloned into the pTOPO-Blunt Simple vector. A no-DNA control was included. All the inserts were directly sequenced in both directions (TsingKe Biological Technology, Wuhan).

### Bioinformatic analyses

The nucleotide sequence of *Hc-tgfbr2* was aligned with the coding and genomic sequences available for *H*. *contortus* [23, 24]. To confirm the identity of the genes isolated, the coding sequence of *Hc-tgfbr2* was compared with the publicly available sequences in non-redundant databases using BLASTx from NCBI (http://www.ncbi.nlm.nih.gov/BLAST). All coding sequences were conceptually translated into predicted amino acids (aa) using DNAstar software (http://www.dnastar.com/). The predicted amino acid sequence of *Hc-*TGFBR2 and its homologues were aligned using the program Clustal W [25], and the alignment adjusted manually. Functional domains were identified and highlighted using the program Photoshop CS 6.0.

For phylogenetic analysis, the predicted amino acid sequences of *Hc-*TGFBR2 and 14 other homologues were aligned. Homologous sequences from 14 species, retrieved from the GenBank database, represented *Ascaris suum* (ADY40134.1) [26], *Brugia malayi* (CDQ03802.1) [27], *Caenorhabditis brenneri* (CAP28808.1) [28], *C*. *elegans* (CCD63118.1) [4], *C*. *remanei* (EFO86570.1) [29], *Danio rerio* (AAD19844.1) [30], *Drosophila melanogaster* (AAF55079.1) [31], *Homo sapiens* (NP_001097.2) [32], *Loa loa* (EFO28480.2) [33], *Mus musculus* (NP_031423.1) [34], *Ovis aries* (AFS17245.1) [35], *Toxocara canis* (KHN86439.1) [36], *Wuchereria bancrofti* (EJW87572.1) [33] and *Xenopus laevis* (AAB00480.1) [37]; a TGF-β type I receptor of *C*. *elegans* (CCD62175.1) [5] was used as an outgroup (Table 1). Phylogenetic analyses were conducted separately using the neighbor-joining (NJ), maximum parsimony (MP) and maximum likelihood (ML) methods, respectively, based on the Jones-Taylor-Thornton (JTT) model employing the program MEGA v.6.0 [38]. Confidence limits were assessed using a bootstrap procedure with 1,000 pseudo-replicates for NJ, MP and ML trees; other settings were at default values in MEGA v.6.0 [38]. A 50% cut-off value was implemented for the consensus tree.

**Table 1:** Sequences of TGF β type II receptors used for phylogenetic and alignment analysis.

Species	GenBank accession number	Reference
***Ascaris suum***^**1**^	ADY40134.1	[26]
***Brugia malayi***^**1**^	CDQ03802.1	[27]
***Caenorhabditis briggsae***	CAP28808.1	[28]
***Caenorhabditis elegans***^**1**^	CCD63118.1	[4]
***Caenorhabditis elegans***^**2**^	CCD62175.1	[5]
***Caenorhabditis remanei***	EFO86570.1	[29]
***Danio rerio***^**1**^	AAD19844.1	[30]
***Drosophila melanogaster***^**1**^	AAF55079.1	[31]
***Homo sapiens***^**1**^	NP_001097.2	[32]
***Loa loa***	EFO28480.2	[33]
***Mus musculus***^**1**^	NP_031423.1	[34]
***Ovis aries***	AFS17245.1	[35]
***Toxocara canis***^**1**^	KHN86439.1	[36]
***Wuchereria bancrofti***	EJW87572.1	[33]
***Xenopus laevis***^**1**^	AAB00480.1	[37]

^1^ Sequence was used in the alignment.

^2^ Sequence was used as an outgroup in phylogenetic analysis.

### Transcriptional analysis by real-time PCR

Real-time PCR was carried out in a volume of 10 μL using 0.2 μM of each forward primer and reverse primers, SYBR *Premix Ex* Taq II (Tli RNaseH Plus, Takara, Japan), ROX Reference Dye II (Takara, Japan), as recommended by the manufacturer (Takara) employing an AII A7 thermal cycler (Bio-Rad, USA). No-DNA and normalizer (β-tubulin 8–9) controls were included in each PCR run, and 100 ng of cDNA was included in PCR. The following cycling protocol was used: 95°C for 30 s, followed by 40 cycles at 95°C for 15 s, 60°C for 15 s and 72°C for 20 s.

Transcription levels of the *Hc-tgfbr2* gene were assessed three times (in triplicate) in each of eight developmental stages (i.e. egg, L1, L2, L3, female L4, male L4, female adult and male adult) of *H*. *contortus* (Haecon-5 strain) using the primers Hc-tgfbr2-rtF/R (S1 Table). β-tubulin 8–9 was used as a normalizer, employing specific primers Hc-tub-rtF/R (S1 Table) [39]. PCR efficiency was calculated using an established formula, and the data of the real-time PCR were analysed to compare with the relative transcription levels in an egg (egg = 1) of *H*. *contortus* using the 2^-ΔΔCt^ method [40]. A one-way ANOVA was used in the statistical analysis; *P* < 0.05 and *P* < 0.01 were set as the criterion for statistical significance; each *P*-value was determined by one-to-one comparisons. Statistical differences at 0.05 and 0.01 levels were represented by lowercase letters and capital letters, respectively.

### Detection of expression patterns in *H*. *contortus* by immunohistochemistry

The nucleotide sequences encoding the truncated protein *Hc-*TGFBR2 (290–642 aa) were PCR-amplified from a plasmid containing the sequence coding for *Hc-tgfbr2* in a volume of 20 μL using 1 ng of plasmid, 0.2 μM of each forward primer and reverse primers (Hc-tgfbr2-eF/eR, S1 Table) and *Es* Taq Master Mix (1 U Taq DNA polymerase, Beijing ComWin Biotech Co. Ltd., China), as recommended by the manufacturer (ComWin), employing the following protocol: 95°C/3 min, followed by 95°C/30 s, 60°C/40 s; 72°C/1 min for 35 cycles; and 72°C/4 min. The amplicon was inserted into the expression vector pET-28a, and the construct was transformed into *E*. *coli* BL21-CodonPlus (DE). Then, the truncated protein, *Hc-*TGFBR2 (290–642 aa), was expressed and purified (Friendbio Science & Technology Co. Ltd, Wuhan, China). All proteins were analyzed by SDS-PAGE.

Prior to the immunization, a pre-bleed was taken from each rabbit, and ‘negative’ serum prepared. Then, two rabbits were injected subcutaneously at four sites (both sides of the spine, neck, abdomen and back) with 500 μg of purified protein in Freund’s Incomplete Adjuvant (three immunizations at two-week intervals). A bleed was taken from each rabbit at the fifth week to assess the anti-*Hc*-TGFBR2 antibody titer. Two weeks later, the rabbits were immunized for the fourth time, and a final bleed was taken one week after the last immunization, and was designated the ‘positive’ serum. Sera were prepared according to a standard procedure [41]. Both positive and negative control sera were assessed (at 1/500 dilution) on a Western blot of proteins extracted from adults of *H*. *contortus* using the Total Protein Extraction Kit (Bestbio Co., China).

Approximately 50–100 *H*. *contortus* adults harvested from abomasa from infected goats were washed five times in 50 ml physiological saline, and fixed in 4% paraformaldehyde (Biosharp, China) at 4°C for 3 days. Single female and male worms were dehydrated separately in a graded ethanol series (75% for 4 h, 85% for 2 h, 90% for 2 h, 95% for 1 h each and 100% two times for 30 min) and embedded in paraffin. Sections (4 μm) were cut using a microtome and mounted on to polysine slides. Paraffinated sections (xylene-treated two times for 20 min) were rehydrated in a graded ethanol series (100% two times for 10 min; 95% once for 5 min, 90% once for 5 min, 80% once for 5 min, 70% once for 5 min), and then washed three times (5 min each) in phosphate-buffered saline (PBS). A microwave was used to recover antigens, and hydrogen peroxide (3%) was used to reduce the non-specific staining by endogenous catalase [42]. Slides were washed three times with PBS (5 min each), and blocked with 5% w/v bovine serum albumin (BSA) for 20 min in a humidified chamber. A volume of 50 μl of the ‘positive’ or ‘negative’ sera (each at 1:100 dilution) was incubated at 4°C overnight, respectively. Sera were removed and slides were washed three times with PBS (5 min each), followed by incubation in the dark at 37°C for 50 min in sheep anti-rabbit immunoglobulin (IgG; 1/500 dilution) conjugated with fluorescein (Aspen, China). This secondary antibody was removed, and slides were washed three times with PBS (5 min each), followed by an incubation at 24°C for 5 min in 4,6-diamidino-2-phenylindole (DAPI) solution in the dark. The sections were washed again in the same way, and then examined using an epifluorescence microscope (Olympus CX-21, Japan). Images were processed using Photoshop CS 6.0.

### RNA interference in *H*. *contortus*–preparation and implementation

Two pairs of specific primers (Hc-tgfbr2-sF1/sR1 and Hc-tgfbr2-sF2/sR2) were designed to PCR-amplify the coding sequence of the proposed functional domain (1724 bp) of *Hc-*TGFBR2 from the plasmid containing the *H*. *contortus Hc-tgfbr2*-coding sequence for the subsequent construction of two plasmids used for the synthesis of specific dsRNAs (S1 Table). Primers designed to produce the antisense single-stranded RNA (antisense ssRNA) were tagged with a T7 promoter site in the forward direction (Hc-tgfbr2-sF1) and a *Bam*H I restriction enzyme cleavage site in the reverse direction (Hc-tgfbr2-sR1). Other primers designed to produce the sense single-stranded RNA (sense ssRNA) were tagged with a *Bam*H I restriction enzyme cleavage site in the forward direction (Hc-tgfbr2-sF2) and a T7 promoter site in the reverse direction (Hc-tgfbr2-sR2). The cycling protocol for the two amplifications of the 1724 bp *Hc-*TGFBR2 domain was: 95°C/5 min, followed by 95°C/30 s, 55.4°C/30 s; 72°C/2 min for 35 cycles; and 72°C/5 min. The PCR amplifications were performed in a volume of 50 μL using 1 ng of plasmid, 0.4 μM of each forward primer and reverse primers (S1 Table), 0.2 mM each of dNTP and 1 U Phanta Super-Fidelity DNA Polymerase (Vazyme Biotech Co Ltd, China), as recommended by the manufacturer (Vazyme). Then, the two amplicons were cloned separately into the pTOPO-Blunt Simple vector (Aidlab Biotechnologies Co. Ltd.) using the ClonExpress II One Step Cloning Kit (Vazyme Biotech Co., Ltd).

A *cry1Ac* gene from *Bacillus thuringiensis* (*Bt-cry1Ac*, GenBank accession no. GU322939.1), showing no significant homology to any *H*. *contortus* gene, was included as an irrelevant control [21]. The two sequences of *Bt-cry1Ac* designed to produce the antisense and sense ssRNAs were PCR-amplified from *B*. *thuringiensis* cDNA using two sets of specific primers (Bt-cry1Ac-sF1/Bt-cry1Ac-sR1 and Bt-cry1Ac-sF2/Bt-cry1Ac-sR2; S1 Table) in a volume of 50 μL using 750 ng of cDNA, 0.4 μM of each forward primer and reverse primers (S1 Table), 0.2 mM each of dNTP and 1 U Phanta Super-Fidelity DNA Polymerase (Vazyme Biotech Co. Ltd., China), as recommended by the manufacturer (Vazyme) using the following cycling protocol: 95°C/5 min; followed by 35 cycles of 95°C/30 s, 55°C/30 s, 72°C/1 min; and 72°C/5 min, and then inserted into pTOPO-Blunt Simple vector.

All constructs were isolated using the plasmid Maxi Kit (Aidlab Biotechnologies Co. Ltd.), and their amounts were estimated spectrophotometrically (NanoDrop Technologies). Samples were stored at -20°C until use. The constructs containing *Hc-tgfbr2* or *Bt-cry1Ac* fragments were linearised using the restriction enzyme *Bam*HI and then used to synthesize single-stranded RNA (ssRNA) by RNA large-scale T7 production system, according to the instruction manual of MEGAscript T7 transcription kit (Ambion, USA). The quality and yield of linearized templates and ssRNAs were verified by electrophoresis and spectrophotometric analysis (NanoDrop Technologies), respectively. Equal amounts (500 μg) of sense ssRNA and antisense ssRNA were used to synthesize double-stranded RNA (dsRNA) using the manufacturer’s protocol (Ambion, USA). The quality and yield of dsRNAs were also verified by electrophoresis and spectrophotometric analysis (NanoDrop Technologies), respectively. All RNA samples were frozen immediately and stored at -80°C until use.

Double-stranded RNA interference was performed on *H*. *contortus* L3s as described previously [21, 43, 44]. In brief, L3s of *H*. *contortus* were exsheathed in 0.1% sodium hypochlorite/PBS for 30 min at 38°C and then immediately washed (5 min each) twice in sterile PBS (23°C) by centrifugation at 600 ×*g*, followed by four times in PBS (23°C) containing Antibiotic-Antimycotic solution (cat. no. 15240062; Gibco, USA; 2.5 μg/ml of amphotericin, 100 μg/ml of streptomycin and 100 IU/ml of penicillin). After the last wash, xL3s were suspended in Earle’s Balanced Salt Solution (EBSS, Sigma; pH adjusted to 5.2) with Antibiotic-Antimycotic (same concentrations) to final concentration of 33,000 xL3s/ml. Then, 30 μl of xL3 suspension were dispensed into each well of sterile 96-well flat bottomed microplates, and co-incubated with 10 μl of nuclease-free water (blank control), *Bt-cry1Ac* specific dsRNA (irrelevant control) or *Hc-tgfbr2* dsRNA, respectively. Before co-incubation, the nuclease-free water, *Bt-cry1Ac* specific dsRNA or *Hc-tgfbr2* dsRNA were pre-incubated separately with RNasin (8 U) and Lipofectin Reagent (Invitrogen) for 10 min at 24°C. The final concentration of dsRNA was 1 μg/μL and the co-incubation was at 37°C and 20% CO_2_ for 24 h. Larvae (n = 300) were transferred to 100 μl of fresh EBSS (in triplicate) to incubate for 7 more days, and the numbers of L3s and L4s in the replicate cultures were counted by microscopy, as described previously [21]. In addition, the remaining larvae were collected for RNA extraction and subsequent assessment of transcription of *Hc-tgfbr2* by real-time PCR. The *Hc-*18S gene was used as a reference for normalization [45]. The two sets of primers (Hc-tgfbr2-rtF/Hc-tgfbr2-rtR and Hc-18s-rtF/ Hc-18s-rtR) used in real-time PCR are shown in S1 Table. Real-time PCR was carried out in a volume of 10 μL using 100 ng of cDNA, 0.2 μM of each forward primer and reverse primers (S1 Table), SYBR *Premix Ex* Taq II (Tli RNaseH Plus, Takara, Japan), ROX Reference Dye II (Takara, Japan), as recommended by the manufacturer (Takara) using the following cycling protocol: 95°C for 30 s, followed by 40 cycles at 95°C for 15 s, 60°C for 15 s and 72°C for 20 s. Real-time PCR efficiency was calculated using an established formula, and the data were subjected to analysis using the 2^-ΔΔCt^ method (cf. [40]). All experiments were repeated three times on different days.

## Results

### Characterisation of cDNA, and phylogenetic analysis of amino acid sequence data

**Fig 1:**
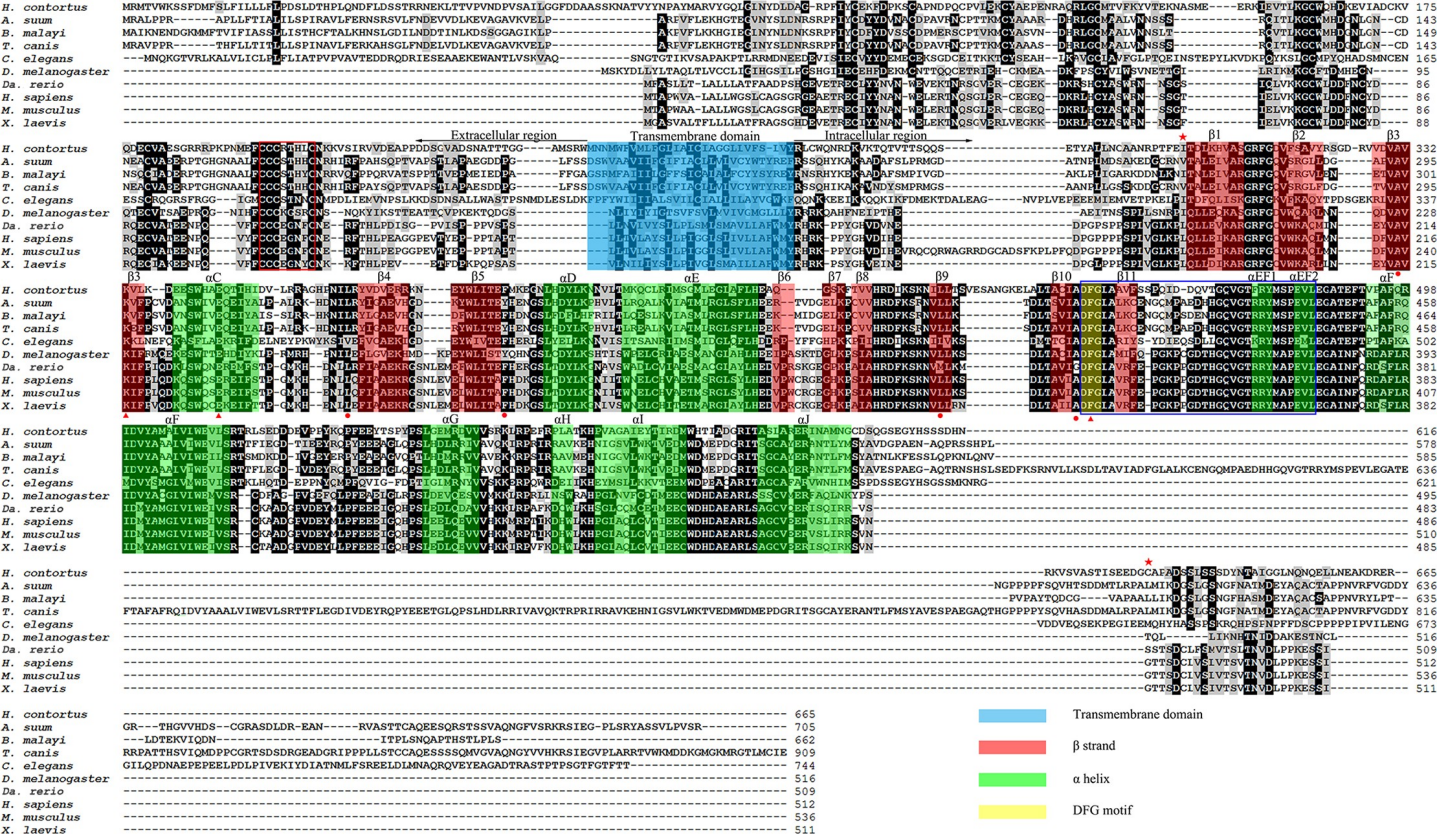
Alignments for *Haemonchus contortus Hc*-TGFBR2 with other homologues of TGF-β type II receptors. Transmembrane domain is marked in blue, β strands are in red, α helices are marked in green and DFG motif is marked in yellow, the characteristic cluster (CXCX_4_C) of TGF-β type II receptors is boxed in red, the protein kinase domain is shown between the sequences marked with the two red stars below. In the protein kinase domain, the activation loop is boxed in blue, the conserved residues involved in forming the hydrophobic pocket are shown as red dots below the sequences and the conserved residues involved in forming water-bridged hydrogen bond are shown as red triangles below the sequences.

The coding sequence of *Hc-tgfbr2* (GenBank accession no. MH829595) is 1998 bp long and encodes 665 amino acids (aa). The predicted protein *Hc-*TGFBR2 has three conserved regions in the TGF-β type II receptor, consistent with functional domains. The sequence of the extracellular region is shorter than that of the intracellular region and has several conserved cysteine residues and a characteristic cluster (CXCX_4_C; boxed in red in Fig 1) near the transmembrane domain of members of the TGF-β type II receptor-family. The sequence of the intracellular region is longer than that of extracellular region and has a conserved serine/threonine kinase domain (Fig 1). Interestingly, the sequences of kinase domain from the TGF-β type II receptors of *H*. *contortus* and four selected nematodes (*A*. *suum*, *B*. *malayi*, *C*. *elegans* and *T*. *canis*) are less conserved than sequences of homologues of five selected non-nematodes (*D*. *melanogaster*, *Da*. *rerio*, *H*. *sapiens*, *M*. *musculus* and *X*. *laevis*). In addition, compared with homologues of other species included here in the analysis, the sequence of the kinase domain of the TGF-β type II receptor of *C*. *elegans* (*Ce*-DAF-4) is most distinct from homologues of four selected nematodes and five selected non-nematodes (Fig 1). A conserved DFG motif, marking the N-terminal end of the activation loop (boxed in blue in Fig 1), was present between the β 10 and β 11 strands of all selected TGF-β type II receptor homologues. Three residues of *Hc*-TGFBR2 (Lys333 from the β3 strand, Glu344 from αC-helix and Phe455 from DFG motif, marked with a red triangle under each amino acid in Fig 1) were the same as the three residues (Lys217, Glu230 and Phe340) of the TGF-β type II receptor of *H*. *sapiens* (*Hs*-ActRIIB) (Fig 1), which form a water-bridged hydrogen bond, instead of salt bridge in the three-dimensional structure of *Hs*-ActRIIB [46]. In addition, the kinase domain of *Hc-*TGFBR2 has conserved side chains of Ala331, Leu361, Phe379, Leu434 and Ala453 (marked with a red dot under each amino acid in Fig 1), which form a hydrophobic pocket for the adenine moiety of ATP in TGF-β type II receptor of *H*. *sapiens* (*Hs*-ActRIIB) [46].

**Fig 2:**
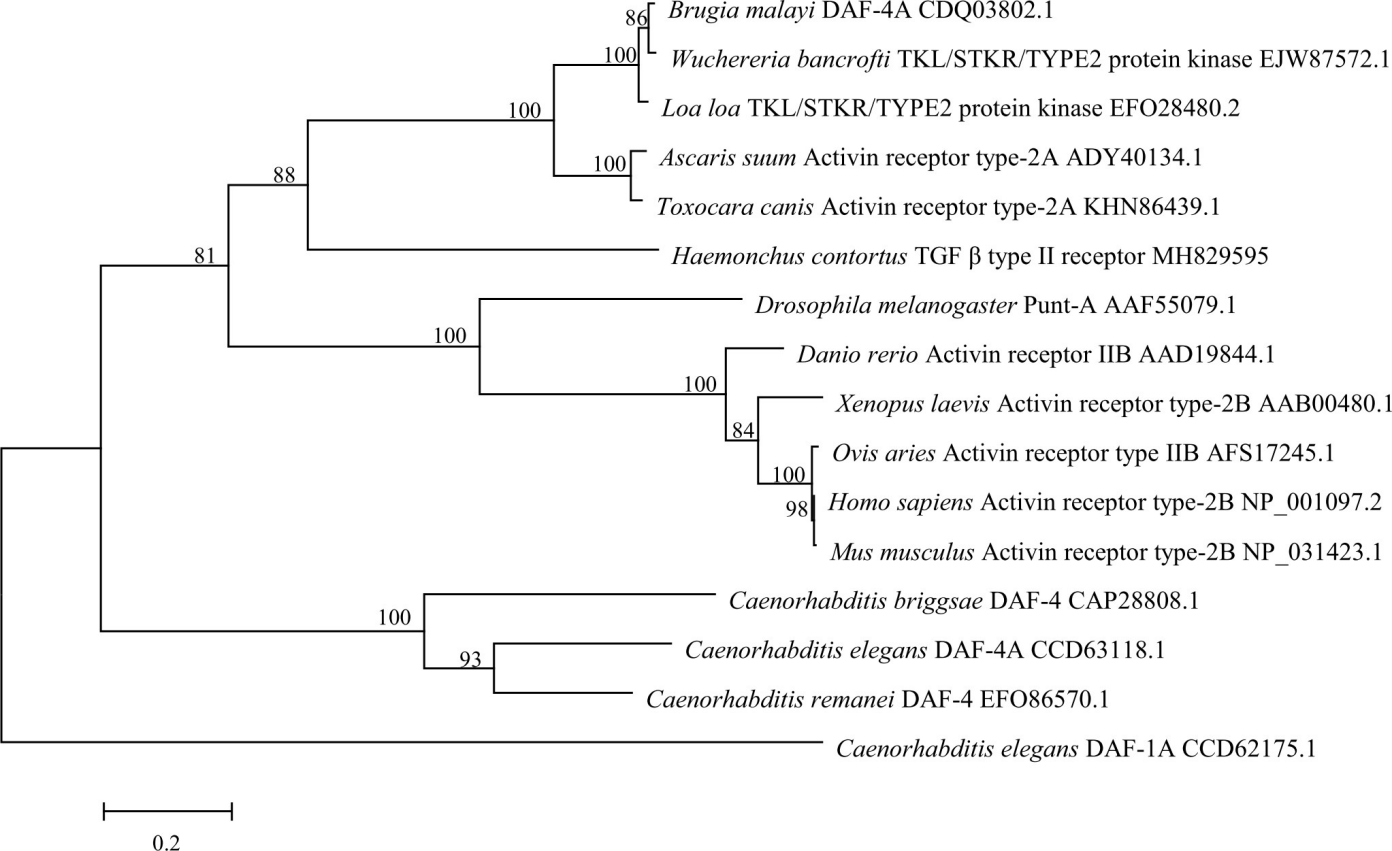
Phylogenetic relationship of *Haemonchus contortu*s *Hc*-TGFBR2 with the TGF-β type II receptors of eight nematodes and six metazoan, whose sequences are listed in Table 1. A TGF-β type I receptor of *Caenorhabditis elegans* (GenBank accession no. CCD62175.1) was set as an outgroup. The Jones-Taylor-Thornton (JTT) model was used employing the MEGA program v.6.0. Nodal support values were shown above or below the branches.

Phylogenetic analyses revealed that *Hc-*TGFBR2 from *H*. *contortus* and other TGF-β type II receptor homologues from five parasitic nematodes represented a cluster with 88% nodal support (Fig 2). In this cluster, TGF-β type II receptor homologues from two roundworms (*A*. *suum* and *T*. *canis*) grouped together with 100% nodal support, whereas TGF-β type II receptor homologues from three filariae (*B*. *malayi*, *W*. *bancrofti* and *L*. *loa*) grouped together with 100% support, and these two small clusters grouped together with absolute support (100%), to the exclusion of *Hc-*TGFBR2 from *H*. *contortus* (Fig 2). Another cluster represented TGF-β type II receptor homologues representing six metazoans (*D*. *melanogaster*, *Da*. *rerio*, *H*. *sapiens*, *M*. *musculus*, *O*. *aries* and *X*. *laevis*) with 100% nodal support, which grouped together with the cluster containing six TGF-β type II receptor homologues of parasitic nematodes (81% support) (Fig 2). The DAF-4 homologues of *Caenorhabditis* spp. grouped together, with 100% nodal support (Fig 2).

### Genomic organisation of *Hc-tgfbr2*

**Fig 3:**
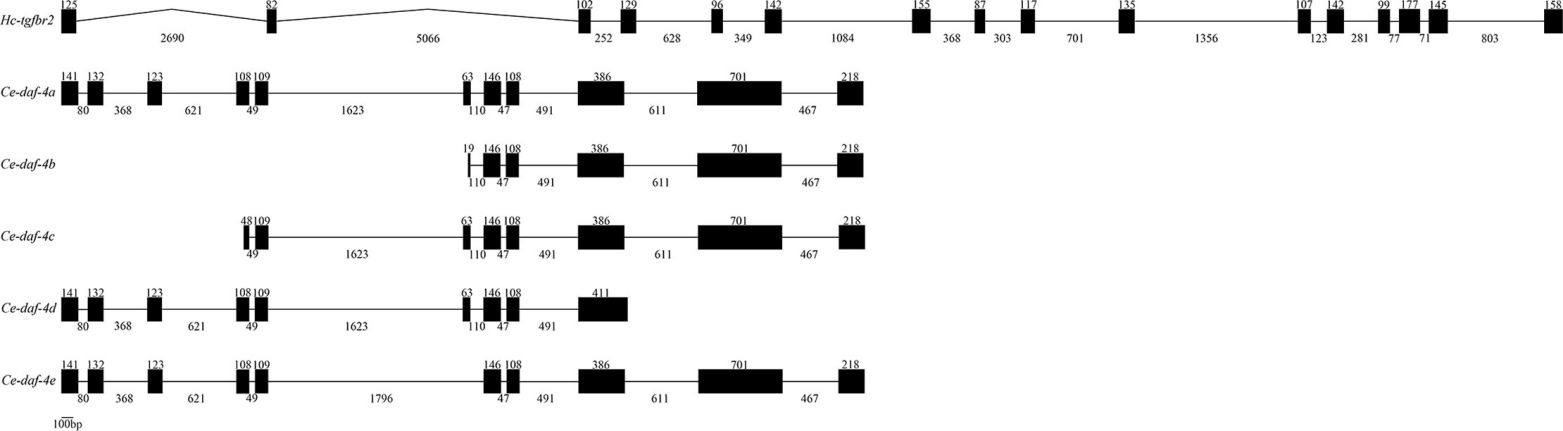
Gene structures of TGF-β type II receptor homologues from *Haemonchus contortus* (*Hc-tgfbr2*) and *Caenorhabditis elegans* (*Ce-daf-4a*, *Ce-daf-4b*, *Ce-daf-4c*, *Ce-daf-4d* and *Ce-daf-4e*). Black boxes represent exons and the numbers above displayed the lengths of exons. Lines between the exons represent introns, and the numbers above indicate the lengths of introns.

The full-length genomic sequence of *Hc-tgfbr2* (GenBank accession no. MH829595) is 16,150 bp long, with 16 exons (82–177 bp) and 15 introns (71–5066 bp), including two introns of 2690 bp and 5066 bp, respectively (Fig 3). Compared with *Ce-daf-4*, *Hc-tgfbr2* contains more exons and introns.

### Transcription of *Hc-tgfbr2* in different developmental stages of *H*. *contortus*

**Fig 4:**
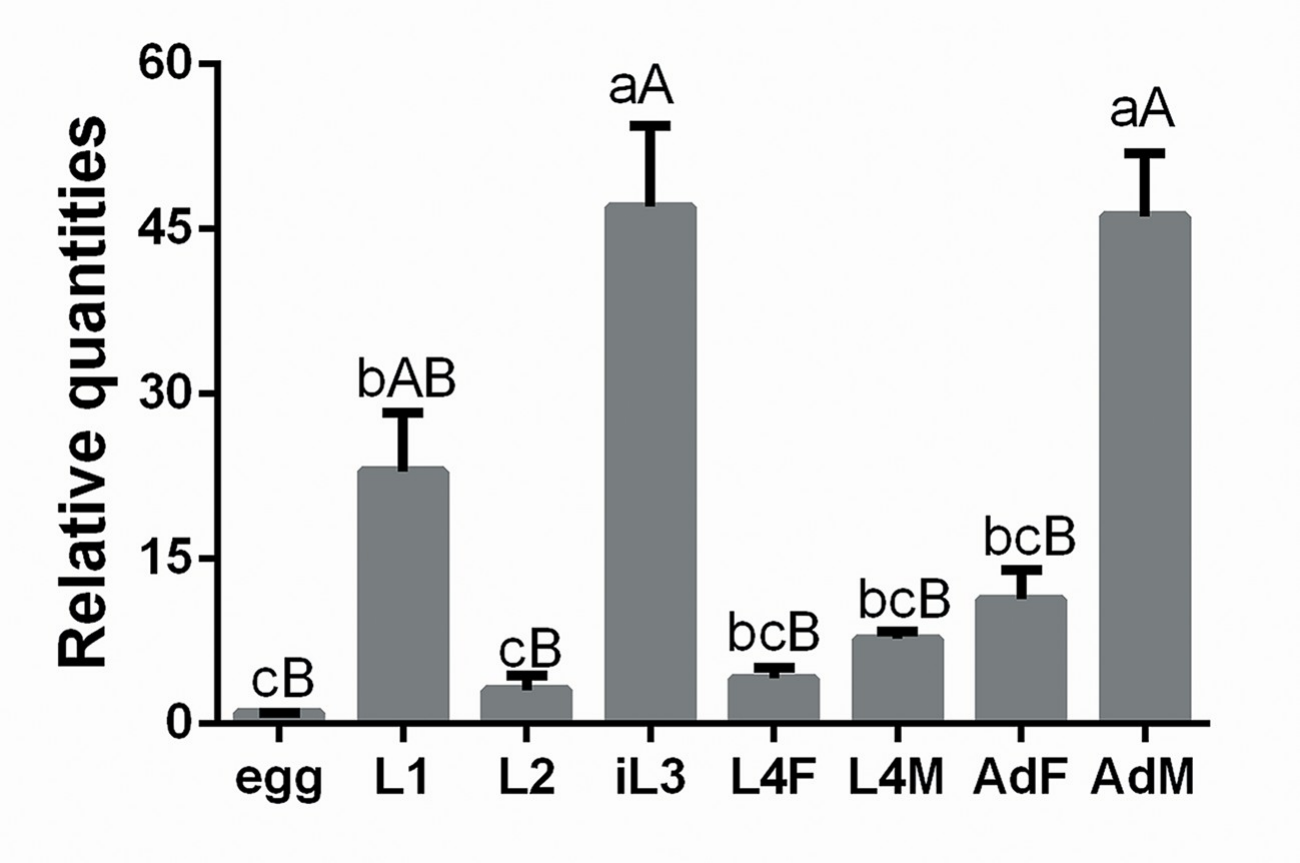
Transcriptional levels of *Hc-tgfbr2* gene in eight developmental stages of *Haemonchus contortus*. The relative abundance of *Hc-tgfbr2* was assessed in eight developmental stages of *H*. *contortus*, including eggs (Egg), first-stage larvae (L1), second-stage larvae (L2), third-stage larvae (L3), females of fourth-stage larvae (L4F), males of fourth-stage larvae (L4M), adult females (AdF) and adult males (AdM). The relative quantities (compared with egg, egg = 1) are shown as mean values (± standard error of the mean, SEM). The significant differences between stages were indicated by different letters while one same letter present between stages indicated no difference. The different lowercase letter (a, b) means *P* < 0.05 while the same lowercase letters mean *P* > 0.05; the different capital letter (A, B) means *P* < 0.01 while the same capital letter indicates *P* > 0.01.

The relative transcription of *Hc-tgfbr2* in eight distinct developmental stages (eggs, L1s, L2s, L3s, female L4s, male L4s, female adults and male adults) of *H*. *contortus* was estimated by real-time PCR, with peak transcription exhibited in L3 and male adult stages (Fig 4). There was no difference in the relative level of *Hc-tgfbr2* transcription between L3s and adult males (*P* > 0.9999). The level of *Hc-tgfbr2* transcription in L3s was significantly higher than that of eggs (*P* < 0.0001), L1s (*P* = 0.0105), L2s (*P* < 0.0001), female L4s (*P* = 0.0002), male L4s (*P* < 0.0001) and female adults (*P* = 0.0002). The level of *Hc-tgfbr2* transcription in adult males was higher than in eggs (*P* < 0.0001), L1s (*P* = 0.0141), L2s (*P* < 0.0001), female L4s (*P* < 0.0001), male L4s (*P* < 0.0001) and female adults (*P* = 0.0003) (Fig 4). In addition, the level of *Hc-tgfbr2* transcription was similar among eggs, L1s, L2s, female L4s, male L4s and female adults (Fig 4).

### Expression pattern of *Hc-tgfbr2* in *H*. *contortus* adults

**Fig 5:**
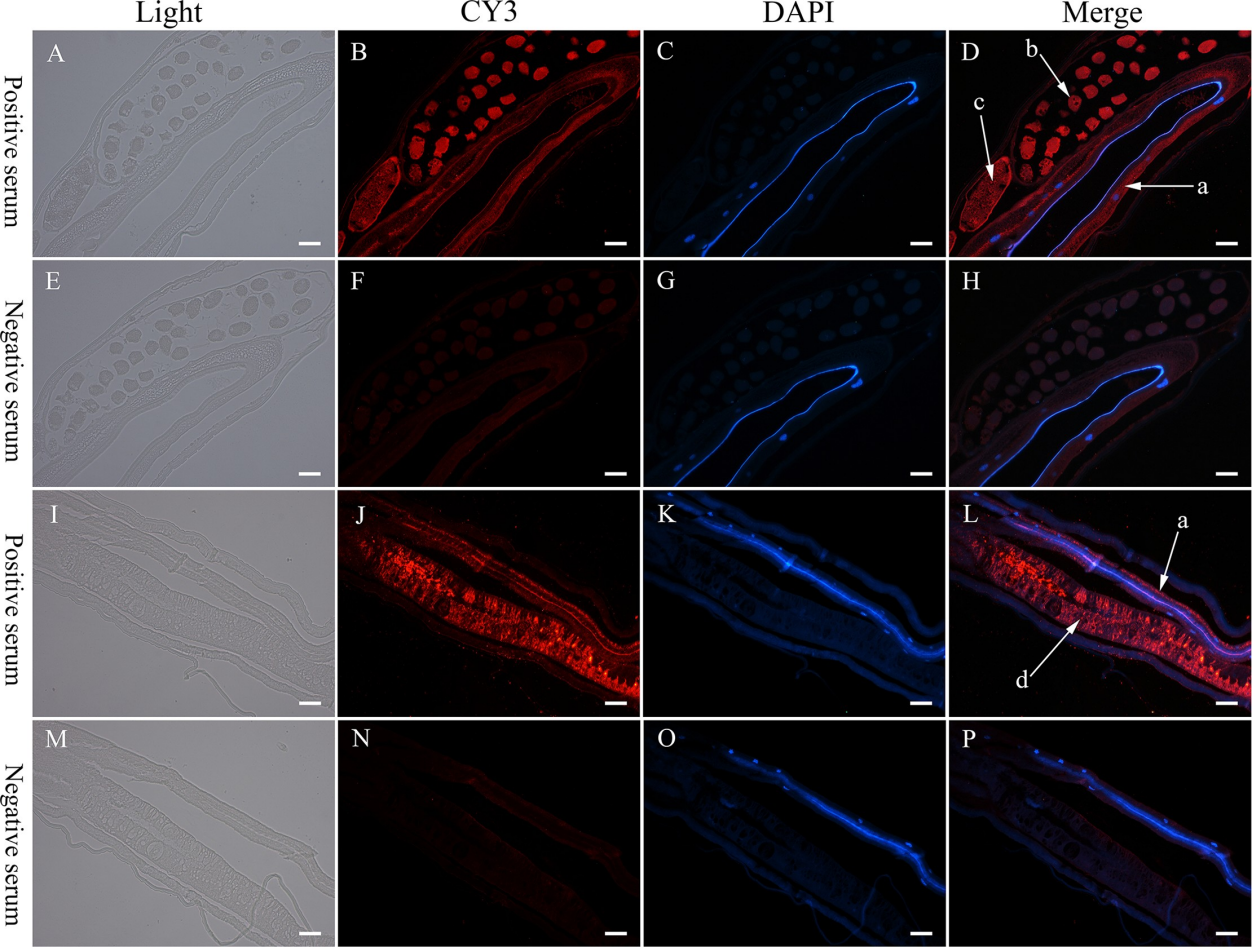
The localization of *Hc-*TGFBR2 in *Haemonchus contortus* adults by immunohistochemistry. (A-H) the localization of *Hc-*TGFBR2 in *H*. *contortus* adult females, (I-P) the localization of *Hc-*TGFBR2 in *H*. *contortus* adult males. The ‘positive’ serum was the serum from the final bleed after the last immunisation (containing the antibody against recombinant *Hc*-TGFBR2); the ‘negative’ serum was the serum from the pre-bleed before the first immunization with recombinant *Hc*-TGFBR2 (exposure time is 2500 ms). a: intestine; b: eggs within the uterus; c: ovaries; d: testes, scale-bar: 50 μm.

The cDNA of truncated *Hc-tgfbr2* (1059 bp), which encodes 353 amino acids (~ 47 kDa), was expressed in *E*. *coli* (S1 Fig), and the purified recombinant protein (S1 Fig) was used to immunize rabbits to produce serum antibody. The polyclonal antibody (positive-serum) from rabbits bound specifically to the native *Hc-*TGFBR2 (74.8 kDa) from *H*. *contortus* adults (S2 Fig). Using this antibody as a probe, *Hc-*TGFBR2 was found to be highly expressed in the intestine, ovaries and eggs within the uterus of female adults (Fig 5A–5H). In adult males, *Hc-*TFGBR2 was expressed in the intestine and testes (Fig 5I–5P).

### Assessment of the effect of *Hc-tgfbr2*-specific dsRNA on larval development of *H*. *contortus*

**Fig 6:**
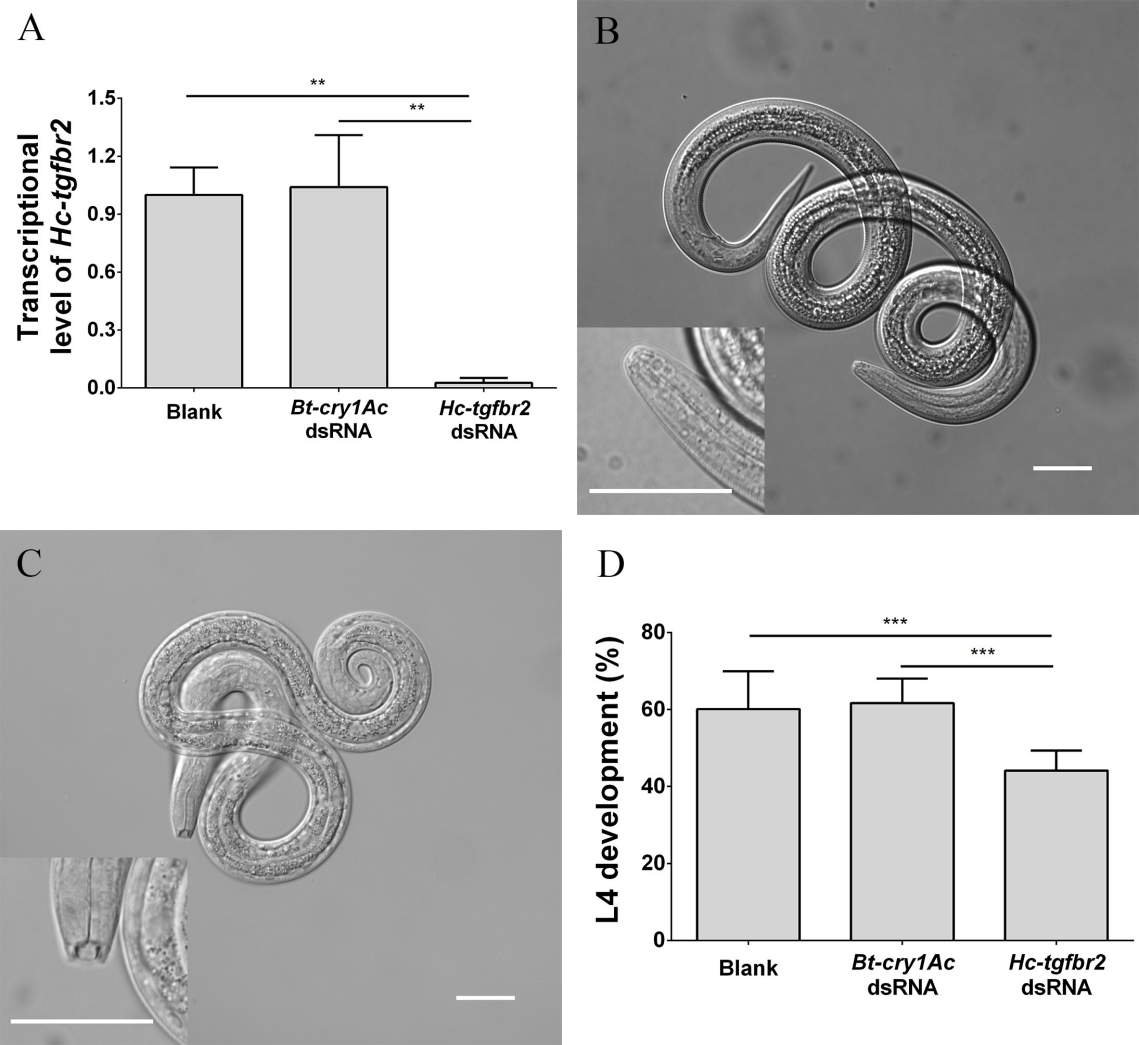
Effects of *Hc-tgfbr2* dsRNA-treatment on *Haemonchus contortus* development. (A) *Hc-tgfbr2* transcription in *H*. *contortus* following RNAi assessed by RT-PCR. (B) Larvae at the xL3 stage. The inset shows the enlarged view of the buccal region. (C) Larvae at the L4 stage. The inset shows the well-developed and functional mouth (buccal cavity) (D) The number of L4s that developed in vitro for another 7 days after RNAi. Scale-bar: 25 μm; * means *P* < 0.05; ** means *P* < 0.01 and *** means *P* < 0.001.

Real-time PCR results showed that the transcription of *Hc-tgfbr2* decreased significantly following the soaking of xL3s of *H*. *contortus* in *Hc-tgfbr2* dsRNA for 24 h, compared with no-dsRNA template and irrelevant dsRNA controls (*Bt-cry1Ac* dsRNA) (*P* = 0.0015 and *P* = 0.0012, respectively); the transcription of *Hc-tgfbr2* was similar between the two control groups (Fig 6A). In addition, fewer xL3s developed to L4s in the *Hc-tgfbr2* dsRNA-treated group compared with the two control groups (*P* < 0.0001), and there was no difference in L4 development between the two control groups (Fig 6D).

## Discussion

In this present study, we isolated and characterised a gene (*Hc-tgfbr2*) that encodes a TGF-β type II receptor homologue in *H*. *contortus*. Of the diverse sequences representing the extracellular region of 10 selected TGF-β type II receptor homologues, *Hc-*TGFBR2 contained a conserved and characteristic feature (CXCX_4_C), which is known as the key determinant of ligand-binding specificity [47], indicating that *Hc-*TGFBR2 can specifically bind the TGF-β ligand. Many kinases contain an αC-helix, which is close to the active site and interacts with essential and conserved kinase elements. This structural characteristic appears to be essential for kinase regulation [48]. In the structure of *Hs*-ActRIIB from *H*. *sapiens*, the interactions between such an αC-helix and the three specific structures, including the hydrophobic pocket, the water-bridged hydrogen bond and the DFG motif, contribute to the unphosphorylated activation loop to adopt an active conformation [46]. Compared with *Hs*-ActRIIB, *Hc-*TGFBR2 has the αC-helix and conserved residues involved in forming these three specific structures (Fig 1); thus, we propose that unphosphorylated activation loop of *Hc-*TGFBR2 is an active conformation that can phosphorylate the TGF-β type I receptor (*Hc*-TGFBR1) to transmit signals.

Phylogenetic analysis revealed that *Hc-*TGFBR2 and five TGF-β type II receptor homologues from parasitic nematodes are genetically closer to the TGF-β type II receptor (activin receptor type IIB, *Hs*-ActRIIB) of *H*. *sapiens* than the TGF-β type II receptor (*Ce*-DAF-4) of *C*. *elegans*, which is consistent with previous results for the TGF-β type I receptor *Hc-tgfbr1* [21]. However, compared with *C*. *elegans*, key molecules involved in the insulin-like signalling pathway of *H*. *contortus*, including phosphoinositide 3-kinases, insulin-like receptor kinase, phosphoinositide-dependent protein kinase-1 and the fork head transcription factor, were more conserved than those in the TGF-β signalling pathway (encoded by *Hc-tgfbr1* and *Hc-tgfbr2*) [21, 49–52].

In the present study, the *Hc-tgfbr2* gene was shown to be transcribed at a higher level in L3s of *H*. *contortus* than other developmental stages, except for male adults (Fig 4), suggesting that *Hc-tgfbr2* is also a key factor regulating development (the transition from free-living stage to the parasitic stage) in *H*. *contortus*. In *C*. *elegans*, *Ce-daf-4* is the sole TGF-β type II receptor involved in the TGF-β signaling pathway, including the DBL-1 signalling and DAF-7 signalling pathways [2, 4]. The DAF-7 signalling pathway mainly regulates dauer formation [2], whereas the DBL-1 signalling pathway has been shown to regulate body size and male-tail morphology in *C*. *elegans* [53, 54]. Here, *Hc-tgfbr2* was transcriptionally up-regulated in male adults of *H*. *contortus* (Fig 4); we found only one TGF-β type II receptor homologue in *H*. *contortus* genome in the NCBI database using BLASTx, suggesting that *Hc-tgfbr2* encodes the sole TGF-β type II receptor involved in both DBL-1 and DAF-7 signalling pathways in *H*. *contortus*. However, in *H*. *contortus* adult males, *Hc-tgfbr2* may function in spermatogenesis and other reproductive processes. This aspect requires further investigation.

In *C*. *elegans*, the DAF-7 signalling pathway regulates dauer or reproductive development, depending on the different cues from the environment sensed by the nerves [5, 6, 55, 56]. The TGF-β type II receptor *Ce-daf-4* is highly expressed in the nervous system of *C*. *elegans* [5]. However, this was not the case for *Hc-tgfbr2* in *H*. *contortus*, where it was expressed in the intestine as well as the ovaries (and eggs) within the uterus of females and the testes of males of adult *H*. *contortus* (Fig 5), suggesting that *Hc*-TGFBR2 functions in adults and is reliant on the intestine and gonad. The intestine of *H*. *contortus* is a prominent source for mucosal antigens [57], and the DBL-1 pathway can regulate innate immunity in *C*. *elegans* [58, 59]. It is reported that Foxp3^+^ regulatory T cells can act against immune responses induced by infection [60], and the TGF β pathway takes apart in the parasite-driven inhibition of host immunity [61]. Recently, a functional TGF-β mimic of the murine parasitic nematode *Heligmosomoides polygyrus* which was not homologous to any members of TGF-β ligand family could bind the host TGF β receptor to induce expression of Foxp3^+^ regulatory T cells in the host [62], suggesting that some members of TGF β superfamily from parasites may take apart in regulation of the host immune system. Taken together, we propose that *Hc*-TGFBR2 might be involved in the immune evasion of parasites by suppressing host immune responses against *H*. *contortus*; this hypothesis is worthy of investigation.

We deduce that *Hc*-TGFBR2 might regulate the reproductive development due to its abundant expression in ovaries (and eggs) within the uterus of females and the testes of males of adult *H*. *contortus* (Fig 5). In *C*. *elegans*, the R-Smad *Ce-*DAF-8 downstream of *Ce-*DAF-4 was identified to function in the adult gonadal distal tip cells and mitotic activity in the germ line [8]. The high transcription level in male adults suggests that *Hc-tgfbr2* might participate in the development of the germline (spermatogenesis and sperm formation).

It is proposed that genes expressed in the tissues accessible to the external environment are accessible to RNAi [44], which was verified by the RNAi results for *Hc-tgfbr1* [21]. Here, the *Hc-tgfbr2* transcription was down-regulated by > 95% following dsRNA treatment, which also supports the viewpoint that RNAi better achieves silencing when conducted on a particular tissue or organ of *H*. *contortus* [44]. The proportion of xL3s that developed to L4s decreased significantly (>25%) following knockdown of the *Hc-tgfbr2* gene by RNAi, indicating that this gene plays a role in regulating the development of *H*. *contortus* from xL3 to L4 in vitro. Furthermore, a TGF-β type I receptor-encoding gene (*Hc-tgfbr1*) has been reported to regulate developmental transition [21]. The findings suggest that the TGF-β signalling pathway can regulate the development of *H*. *contortus*, particularly in the transition from free-living to parasitic stages.

In conclusion, the *Hc-tgfbr2* gene, encoding TGF-β type II receptor, was isolated and characterised for the parasitic nematode *H*. *contortus*. The alignment and phylogenetic analysis indicated that *Hc-*TGFBR2 is a TGF-β type II-like receptor. *Hc-tgfbr2* was transcribed in all developmental stages of *H*. *contortus*, with the highest level in L3s and male adults. Immunohistochemical study indicated that *Hc*-TGFBR2 was expressed in the intestine and gonads of adult stages of *H*. *contortus*. RNAi by specific dsRNA-soaking caused a significant decrease in the transcription of *Hc-tgfbr2* and the development of *H*. *contortus* from xL3 to L4 in vitro. Taken together, these results elucidate a TGF-β type II receptor (*Hc-*TGFBR2) and its association with *H*. *contortus* development, particularly in the transition from the free-living to the parasitic stage.

## Supporting information

S1 Fig Prokaryotic expression and purification of recombinant *Hc-*TGFBR2. (A) prokaryotic expression of recombinant protein *Hc-*TGFBR2 in *E*. *coli* BL21-CodonPlus (DE), as analyzed by SDS-PAGE. M: protein marker; 1: empty vector (pET-28a) induced by 1 mM IPTG; 2: recombinant protein *Hc-*TGFBR2 uninduced; 3: recombinant protein *Hc-*TGFBR2 induced by 1 mM IPTG. (B) purified recombinant *Hc-*TGFBR2, as analyzed by SDS-PAGE. M: protein marker; 1: protein *Hc-*TGFBR2.
(TIFF)

S2 Fig The native *Hc*-TGFBR2 protein from *Haemonchus contortus* adult worm extracts was detected with polyclonal antibody against recombinant *Hc-*TGFBR2 by Western blot analysis. 1: positive serum, the serum from the final bleed after the last immunization (containing the antibody against recombinant *Hc*-TGFBR2) (1:500 dilution); 2: negative serum, the serum from the pre-bleed before the first immunization (without the antibody of *Hc*-TGFBR2) (1:500 dilution).
(TIF)

S1 Table Oligonucleotide primers (5’-3’) used in the present study. (DOCX)
