# Supplementary figures and images for: A TGF-β type II receptor that associates with developmental transition in Haemonchus contortus in vitro
Source: PLoS Negl Trop Dis. 2019 Dec 2;13(12):e0007913. doi: 10.1371/journal.pntd.0007913 (PMC6938378; doi:10.1371/journal.pntd.0007913)

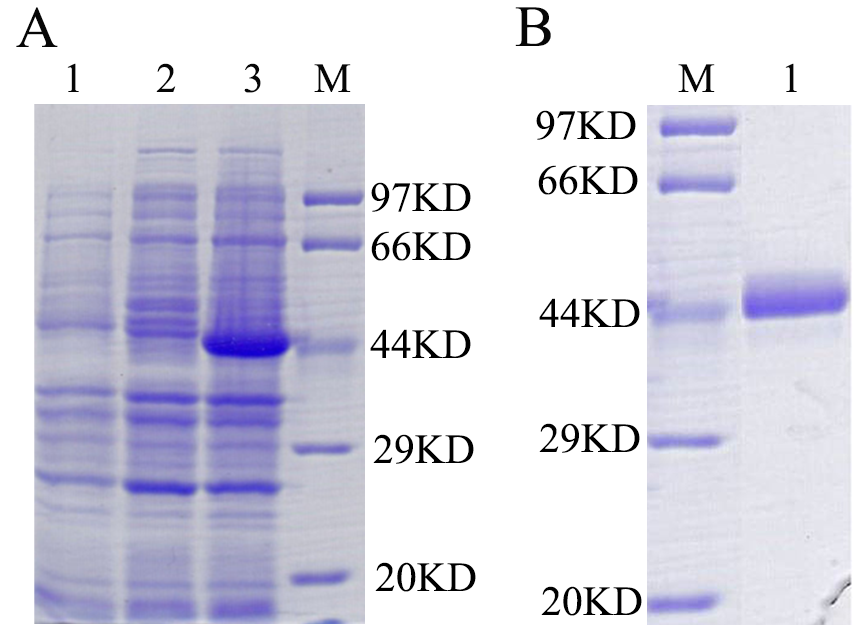

Supplement: S1 Fig — (A) prokaryotic expression of recombinant protein Hc-TGFBR2 in E. coli BL21-CodonPlus (DE), as analyzed by SDS-PAGE. M: protein marker; 1: empty vector (pET-28a) induced by 1 mM IPTG; 2: recombinant protein Hc-TGFBR2 uninduced; 3: recombinant protein Hc-TGFBR2 induced by 1 mM IPTG. (B) purified recombinant Hc-TGFBR2, as analyzed by SDS-PAGE. M: protein marker; 1: protein Hc-TGFBR2. (TIFF) [file pntd.0007913.s001.TIFF]

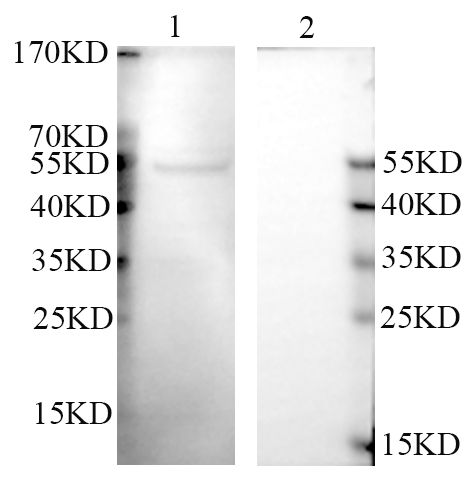

Supplement: S2 Fig — 1: positive serum, the serum from the final bleed after the last immunization (containing the antibody against recombinant Hc-TGFBR2) (1:500 dilution); 2: negative serum, the serum from the pre-bleed before the first immunization (without the antibody of Hc-TGFBR2) (1:500 dilution). (TIF) [file pntd.0007913.s002.tif]
