# Supplementary material for: A TGF-β type II receptor that associates with developmental transition in Haemonchus contortus in vitro
Source: PLoS Negl Trop Dis. 2019 Dec 2;13(12):e0007913. doi: 10.1371/journal.pntd.0007913 (PMC6938378; doi:10.1371/journal.pntd.0007913)
Supplement: S1 Table — (DOCX) [file pntd.0007913.s003.docx]

**S1 Table. Oligonucleotide primers (5’-3’) used in the present study.**

| **Primer** | **Sequence (5’-3’)^a^** |
| --- | --- |
| **Primers for isolating *Hc-tgfbr2* gene and its upstream region** | |
| **Hc-tgfbr2-cF** | ATGAAGTCATTTCGCATTA |
| **Hc-tgfbr2-cR** | TCATGAAAATCGCATACAG |
| **Hc-tgfbr2-gF1** | GGCGATTCTGTTCCTTTCTC |
| **Hc-tgfbr2-gR1** | TCCTAACCGTTACCATCAGC |
| **Hc-tgfbr2-gF2** | GCTGATGGTAACGGTTAGGA |
| **Hc-tgfbr2-gR2** | CAAGCAAGAGTTTCCCATGC |
| **Primers for RNA interference** | |
| **Hc-tgfbr2-sF1** | **TAATACGACTCACTATAGGG**AGAGAATTACGATCTTGACGCCG |
| **Hc-tgfbr2-sR1** | GGATCCCCCTGTCCTTTGCTTCATTC |
| **Hc-tgfbr2-sF2** | GGATCCGAATTACGATCTTGACGCCG |
| **Hc-tgfbr2-sR2** | **TAATACGACTCACTATAGGG**AGACCCTGTCCTTTGCTTCATTC |
| **Bt-cry1Ac-sF1** | **TAATACGACTCACTATAGGG**CCAATACAGTACCAGCTACAG |
| **Bt-cry1Ac-sR1** | GGATCCGATTCGGCTCTCCACAC |
| **Bt-cry1Ac-sF2** | GGATCCCCAATACAGTACCAGCTACAG |
| **Bt-cry1Ac-sR2** | **TAATACGACTCACTATAGGG**GATTCGGCTCTCCACAC |
| **Primers for real-time PCR** | |
| **Hc-tgfbr2-rtF** | ATGCGAATGACGGTGTGG |
| **Hc-tgfbr2-rtR** | ACGGTTGTTAGCTTTTCATTCC |
| **Hc-tub-rtF** | TGTTCCATCACCCAAGGTATCC |
| **Hc-tub-rtR** | TGACAGACACAAGGTGGTTGAGAT |
| **Hc-18s-rtF** | AATGGTTAAGAGGGACAATTCG |
| **Hc-18s-rtR** | CTTGGCAAATGCTTTCGC |
| **Primers for prokaryotic expression** | |
| **Hc-tgfbr2-eF** | CGCGGATCCCTTCTGAACTGTGCAGCCAAT |
| **Hc-tgfbr2-eR** | CCGCTCGAGGTCGCTACTGCTTAAACTGCTA |

^a^ Italics font represent homologous sequences from vectors, underscore represents restriction sites and boldface represent a T7 promoter site.

Hc, *Haemonchus contortus*; Ce; *Caenorhabditis elegans*; Bt, *Bacillus thuringiensis*.
